# Unique Ovarian Metastasis of Small-Cell Lung Cancer and Complete Response After Chemo-Immunotherapy: An Unusual Spread as a Predictor Factor

**DOI:** 10.7759/cureus.65947

**Published:** 2024-08-01

**Authors:** Federico Monaca, Frediano Socrate Inzani, Armando Orlandi, Emilio Bria

**Affiliations:** 1 Medical Oncology, Fondazione Policlinico Universitario Agostino Gemelli IRCCS (Istituto di Ricovero e Cura a Carattere Scientifico), Rome, ITA; 2 Molecular Medicine, Università degli Studi di Pavia, Pavia, ITA

**Keywords:** long-term maintenance, thoracic oncology - areas of interest, extended stage small cell lung cancer (es-sclc), cancer immunotherapy, ovarian metastasis

## Abstract

A 41-year-old woman, never-smoker, accessed the emergency room for an episode of hemoptysis in September 2019. CT scan showed a defect of opacification in the left pulmonary artery and a solid mass of 12 cm in the left annex. PET confirmed high metabolic activity in the ovarian mass and, surprisingly, in the left hilar lung. The patient underwent a left annessiectomy and the histological examination showed a metastasis of small-cell lung cancer (SCLC) that mimicked a primary ovarian cancer. Fibrobronchoscopy and echo-guided biopsy confirmed the diagnosis of pulmonary SCLC. From January 2020, we started systemic therapy with carboplatin, etoposide, and atezolizumab. After six cycles of induction therapy with a complete response, thoracic and prophylactic cranial radiotherapy was done and maintenance therapy with atezolizumab was administered. After 53 months, the patient is still under treatment with a complete radiological response. This case report describes a rare instance of ovarian metastasis from SCLC that responded exceptionally well to immunotherapy. By reviewing literature from 1950 to the present, we identified other cases of ovarian metastases from SCLC, highlighting shared clinical and pathological traits and distinguishing them from primary ovarian tumors. We also examined the potential mechanisms behind the prolonged immunotherapy response observed in this case. As research on SCLC and immunotherapy evolves, this case may offer valuable insights into prognostic and predictive factors for this typically fatal cancer.

## Introduction

Small-cell lung cancer (SCLC) represents about 15% of all lung cancers and it is strongly associated with exposure to tobacco carcinogens; in fact, less than 2% of SCLC cases arise in never-smokers [1]. SCLC has a very high proliferative rate, which brings to an early vascular invasion and, therefore, most patients have metastatic disease at diagnosis, with only one-third of the patients presenting a limited-stage disease when detected. The most frequent sites of metastases are the brain, bone, liver, mediastinal lymph nodes, and adrenal glands [2]. Ovary as a unique metastatic site is a rare occurrence, and an extensive literature search revealed only a few of such documented cases. Even if, recently, it has been shown that chemo-immunotherapy prolongs the overall survival (OS) and the progression-free survival (PFS), the outcome remains very poor with a median OS of about 12 months in patients with advanced SCLC [3]. Here we report a unique case of a never-smoker woman affected by SCLC with unique ovarian metastasis under treatment with chemo-immunotherapy, who is obtaining a durable, complete response for more than 50 months.

## Case presentation

**Figure 1:**
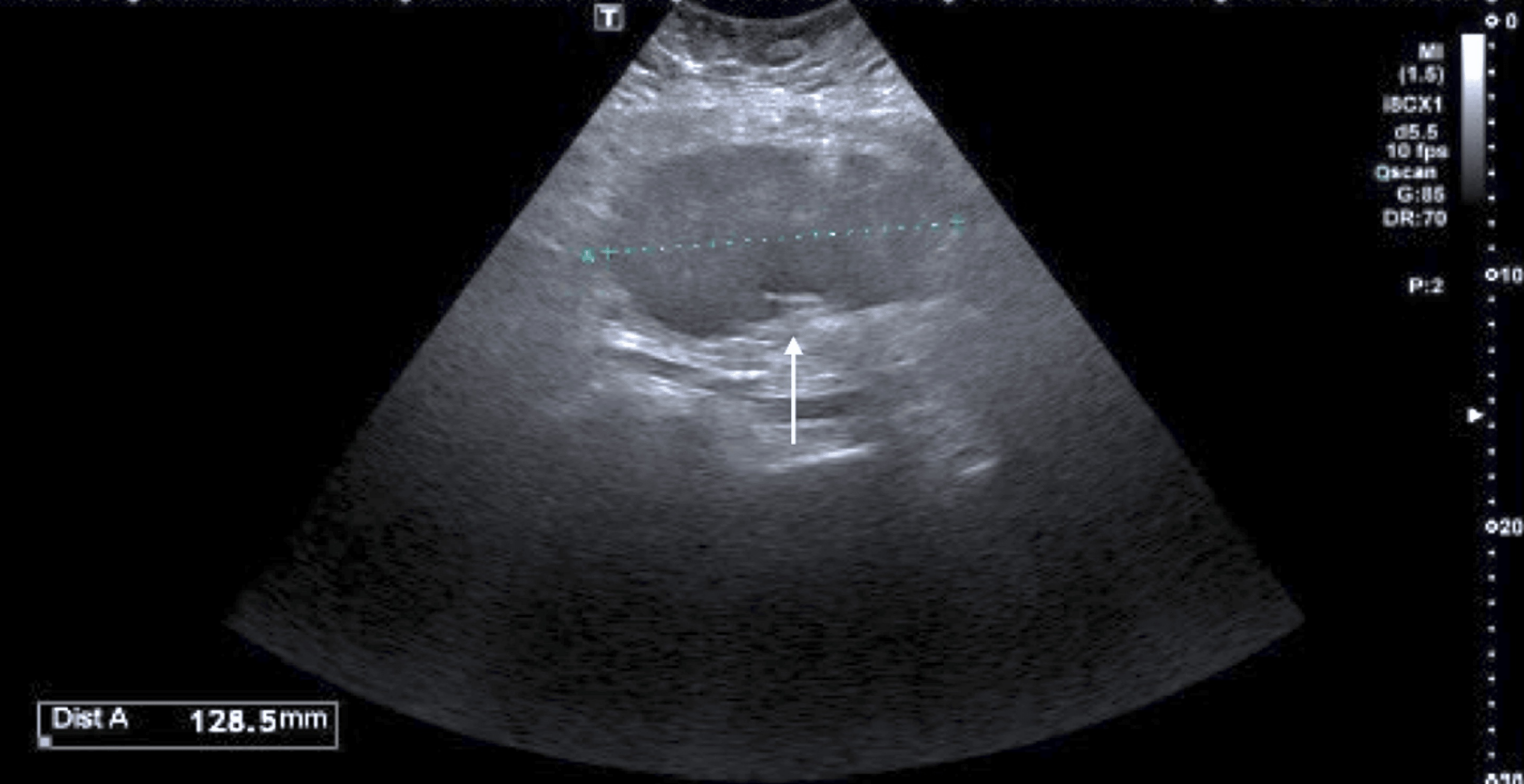
Ovarian ultrasound echography The first ultrasound echography (September 2019) detected left ovarian metastasis with irregular margins (128.5 mm).

We report the case of a 41-year-old woman, a never-smoker, who had two full-term pregnancies, was obese (BMI 43.7), and had a regular menstrual cycle with no other risk factor for ovarian or lung cancer. The patient did not report any weight loss before the diagnosis or episodes of vaginal bleeding or alterations in bowel habits or pelvic pain. She only complained of a persistent cough, which arose one month before, and at the end of September 2019, she was admitted for an episode of hemoptysis. The fibrobronchoscopy (FBS) did not show apparently active bleeding or relevant alterations; cytologic and microbiological exams of bronchoalveolar lavage were negative. The following CT scan showed a defect of opacification in the left pulmonary artery, ascribable to thromboembolism, and a 12 cm lesion in the left annex, not clearly separable from the sigma. Gastroscopy and colonoscopy did not show any mucosal alteration, tumor markers for breast carcinoma (CA 15-3) and gastrointestinal tumors (CEA, CA19-9) were negative, and CA125 and neuron-specific enolase were negative too. Thus, a PET scan was performed and it showed a high metabolic activity of the well-known ovarian mass (SUV max 19.25) and an area of hyperaccumulation in the left hilar pulmonary region (SUV max 12.11). A transvaginal echography confirmed the mass in the left annex, so she underwent a left annessiectomy (Figure 1).

**Figure 2:**
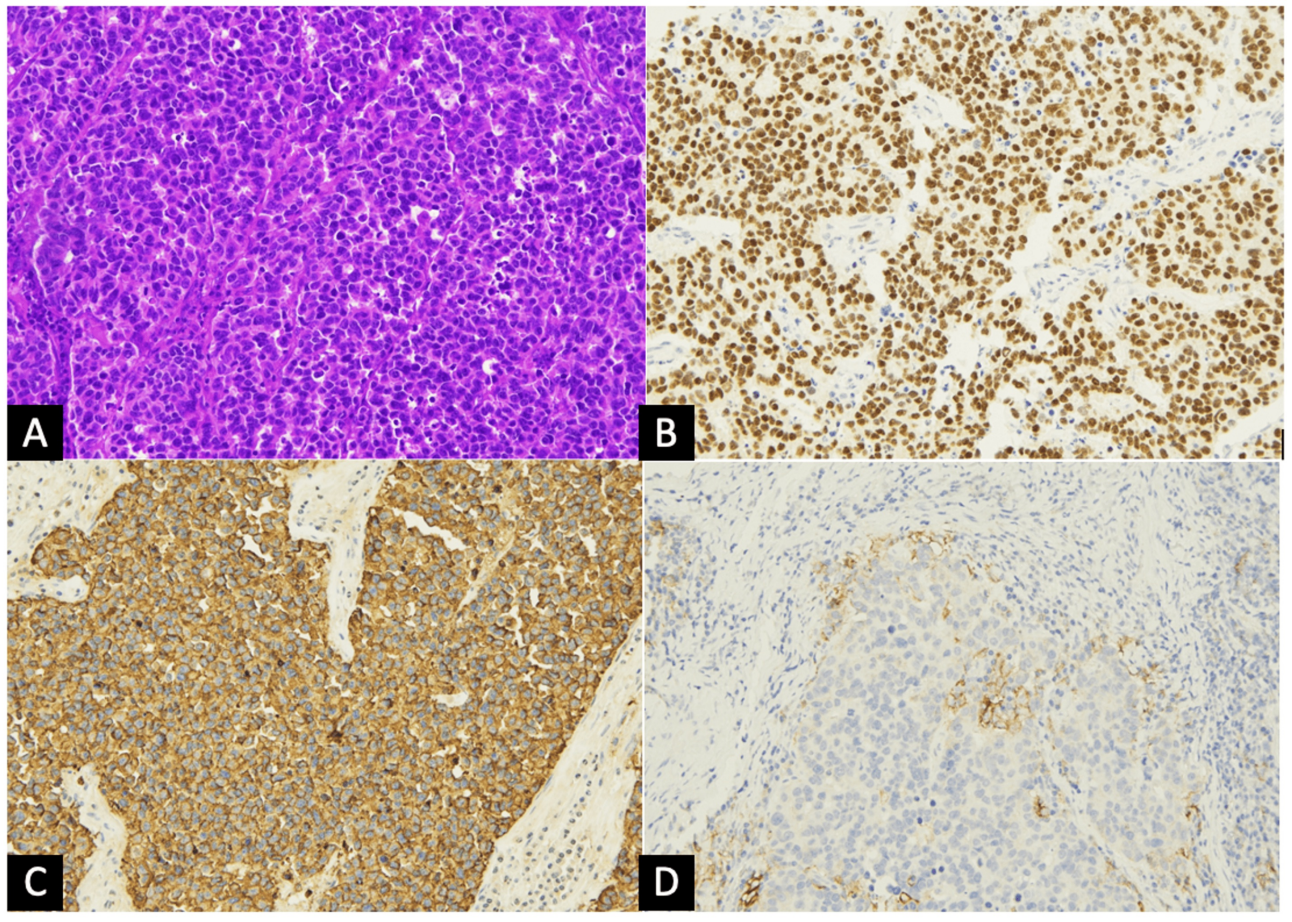
Histological examination of the ovarian mass In the histologic examination, the ovarian mass was characterized by a solid proliferation of epithelioid cells with atypical hyperchromatic nuclei and scant cytoplasm (A, magnification x20) positive at immunohistochemistry for TTF1 (B, magnification x20) and chromogranin A (C, magnification x20) consistent with the diagnosis of metastasis of lung small cell carcinoma. PDL1 is focally, but significantly, expressed by tumor and immune cells (D, magnification x20). Hematoxylin and eosin (A), immunoperoxidase (B-D).

The histological examination revealed a solid proliferation of atypical epitheliomorphic elements and a high nucleus/cytoplasm ratio with necrosis areas. The immunoreactivity test indicated positivity to chromogranin A, synaptophysin, CD56, TTF-1, p53, and cytokeratin 7 and negativity to estrogen, progesterone, WT1, and cytokeratin 20 with a proliferation index (Ki-67) of 70%. The mismatch repair (MMR) proteins evaluation was proficient and PD-L1 expression was 5-10%. These pathological evidences led to the diagnosis of small-cell neuroendocrine carcinoma (“pulmonary type”) (Figure 2). 

**Figure 3:**
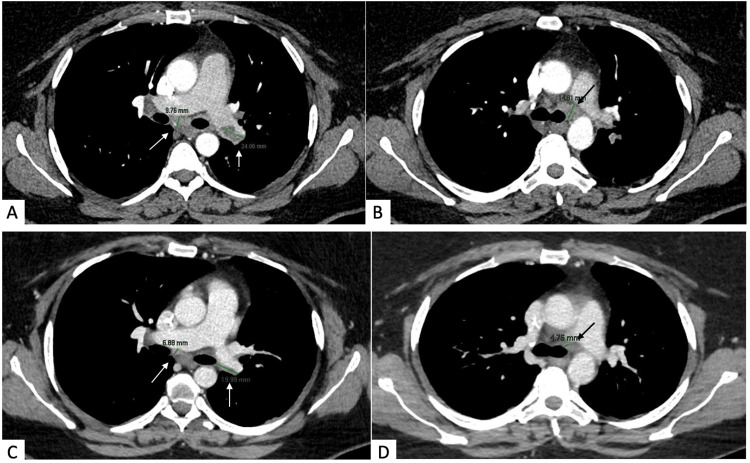
Comparison of pre- and post-treatment CT scans Basal CT scan (January 2020): Image A shows a lesion of 24 mm in the left pulmonary artery (T1, 24 mm) and subcarinal nodes (T3, 10 mm); image B shows the paratracheal left lower nodes (T2, 15 mm). CT scan performed on May 2020: Image C shows the T1 (20 mm) and T3 lesions (5 mm); image D shows the T2 lesion (5 mm).

Subsequently, another FBS was performed in December 2019 and it detected peribronchial tissue between the upper and inferior left lobar bronchus; the biopsy of this tissue confirmed the diagnosis of SCLC (positive for pancitocheratine AE1/AE3, chromogranin A, synaptophysin, and TTF-1). Differentiating between ovarian metastasis of SCLC and primary small-cell carcinomas of the ovary is challenging as both tumors present similar histological and immunohistochemical findings: synaptophysin, chromogranin A, p53, and CD56 are positive for both the tumors while CK20 is negative and TTF-1 is positive in SCLC [4]. The negativity for CK20, the positivity for TTF-1, and the lymphovascular invasion brought us to conclude that it was a primary tumor of the lung with an unusual pattern of ovarian metastasis. Pre-treatment CT scan (January 2020) confirmed a lesion of 24 mm in the left pulmonary artery (T1) with nodes in the paratracheal left lower area (short axis 15 mm, T2), in the subcarinal area (short axis 10 mm, T3), and in the hilar left area (short axis 11 mm) (Figure 3A, 3B). 

In January 2020, the patient started chemo-immunotherapy treatment (carboplatin-etoposide- atezolizumab). Furthermore, given the stability of the lung disease after the induction phase (Figure 3C, 3D), the patient underwent thoracic radiotherapy and prophylactic cranial irradiation thereafter. After more than four years, the patient is still on the maintenance phase with atezolizumab.

## Discussion

We performed a retrospective analysis of case reports about SCLC with ovarian metastases by exploring PubMed and Google Scholar between 1950 and today, using “small cell lung cancer”, “ovarian metastasis,” and “neuroendocrine lung tumors” as keywords. The main characteristics of the 24 case reports are summarized in Table 1.

**Table 1:** A summary of published reports of SCLC with ovarian metastases. CT: chemotherapy; IO: immunotherapy; RT: radiotherapy; NR: not reported; PCI: prophylactic cranial irradiation; CBDCA: carboplatin; AUC: area under the curve; no.: number; cm: centimeters; pos.: positive; neg.: negative.

Author (year)	Age	Smoking history	Clinical presentation	Lesion (cm)	Lesion (monolateral/bilateral)	Lesion (synchronous/metachronous)	Immunochemistry (TTF-1 pos./neg.)	Treatment
Present case	41	No	Hemoptysis	12	Monolateral	Synchronous	TTF-1 positive	CT-IO + lung RT and PCI
Sukumvanich et al. [2] (2005)	42	No	Persistent cough and pleuritic pain	7 and 4	Bilateral	Metachronous	Not reported	CT + lung RT then multiple salvage chemotherapy regimens
Oneda et al. [4] (2020)	72	NR	Vaginal bleeding	6	Monolateral	Synchronous	TTF-1 positive	4 cycles of CBDCA AUC 5
Kitazawa et al. [5] (2019)	42	No	X-ray radiography during controls	6	Monolateral	Metachronous	TTF-1 positive	Multiple CT-RT then lobectomy, RT on mediastinal neoplasm and PCI then multiple CT treatments and finally salpingo-oophorectomy
Garcia et al. [6] (2010)	54	Yes	Persistent cough	5	Monolateral	Metachronous	Not reported	CT + lung RT, PCI then topotecan and finally CBDCA AUC 5
Bing and Adegboyega [7] (2005)	62	Yes	Increased abdominal girth	19	Monolateral	Synchronous	TTF-1 positive	3 cycles of CBDCA
Malviya et al. [8] (1982)	40	Yes	Pelvic discomfort	19	Monolateral	Synchronous	Not reported	Not reported
Varlas et al. [9] (2021)	25	NR	Persistent cough	8.5	Monolateral	Metachronous	TTF-1 positive	CT + lung RT then 6 cycles of CT
Moro et al. [10] (2017)	24	NR	Incidental finding	15 and 2	Bilateral	Synchronous	TTF-1 positive	2 cycles of CT
Barr JS [11] (1953)	39	NR	Persistent cough	7	Monolateral	Synchronous	Not reported	Not treated
Young and Scully [12] Case no. 1 (1985)	26	No	Foul-smelling vaginal discharge	9	Monolateral	2 months before lung tumor	Not reported	RT
Young and Scully [12] Case no. 2 (1985)	42	NR	Incidental finding	10	Monolateral	Synchronous	Not reported	Not reported
Young and Scully [12] Case no. 3 (1985)	40	NR	Abdominal swelling	2.5	Bilateral	Synchronous	Not reported	Not reported
Irving and Young [13] Case no. 1 (2005)	44	NR	Not reported	12 and 26	Bilateral	Metachronous	TTF-1 negative	Not reported
Irving and Young [13] Case no. 2 (2005)	63	NR	Not reported	22 and 0.7	Bilateral	Metachronous	Not reported	Not reported
Irving and Young [13] Case no. 3 (2005)	46	NR	Abdominal swelling	21	Monolateral	Metachronous	TTF-1 positive	Not reported
Irving and Young [13] Case no. 4 (2005)	46	NR	Not reported	NR	Bilateral	Synchronous	TTF-1 negative	Not reported
Irving and Young [13] Case no. 5 (2005)	35	NR	Not reported	9.5 and 7.2	Bilateral	Synchronous	Not reported	Not reported
Irving and Young [13] Case no. 6 (2005)	41	NR	Not reported	NR	Monolateral	Metachronous	TTF-1 positive	Not reported
Irving and Young [13] Case no. 7 (2005)	59	Yes	Not reported	11.5	Monolateral	Synchronous	TTF-1 positive	Not reported
Irving and Young [13] Case no. 8 (2005)	62	Yes	Not reported	NR	Monolateral	Synchronous	Not reported	Not reported
Irving and Young [13] Case no. 9 (2005)	66	NR	Not reported	Multiple 0.5	Monolateral	metachronous	TTF-1 positive	Not reported
Irving and Young [13] Case no. 10 (2005)	62	NR	Respiratory syndrome and virilization	0.15	Monolateral	Synchronous	Not reported	Not reported
Irving and Young [13] Case no. 11 (2005)	71	NR	Pleural effusion	13	Monolateral	Metachronous	Not reported	Not reported

It is very surprising that the median age of diagnosis was 43 years (24-72), whereas in SCLC, it is 68 years, and four out of nine (44%) were never-smokers, which is a very high proportion when compared to that in SCLC where less than 2% of those diagnosed were never-smokers [1]. In most of the cases, metastasis at diagnosis was monolateral (17/24) and oversize (median size 8.75 cm) instead of the typical pattern of metastasis (small and bilateral); in addition, in Oneda et al. [4], the patients had an increased expression of CA125, while in our case, Sukumvanich et al. [2], and in Moro et al. [10], it was normal. Strikingly, TTF-1 is positive in 83% of cases. Irving and Young [13] described a series of 32 cases of lung carcinoma metastatic to the ovary, 14 of which were metastasis of SCLC and, interestingly, case number 8 had a concomitant Sertoli-Leydig tumor, which explains the atypical clinical presentation. Three out of 14 cases had already been published and discussed by Young and Scully [12] in 1985. Table 2 summarizes the cases that reported at least the time of diagnosis of ovarian metastases, the time of progression after the diagnosis, or the time of death/last follow-up. The clinical data of the last two patients were not published as case reports but were extrapolated from a pan-cancer cohort of clinical outcomes with genomics data from more than 25,000 patients treated at Memorial Sloan Kettering Cancer Center [14-16]. 

**Table 2:** A summary of reports where it was possible to calculate the time from treatment of lung cancer, time of progression after diagnosis, or the time of death/last follow-up. NE: not evaluable; no.: number.

Author	Time from treatment of lung cancer (months)	Time of progression after diagnosis of ovarian metastases (months)	Time of death/last follow-up
Present case	NE	NE	53
Sukumvanich et al. [2]	12	18	30
Oneda et al. [4]	NE	NE	12
Kitazawa al. [5]	57	NE	68
Garcia al. [6]	3	5	16
Varlas et al. [9]	16	17	27
Moro et al. [10]	NE	NE	14
Barr [11]	NE	NE	6
Young and Scully [12] Case no. 1	2 months before	2	NE
Young and Scully [12] Case no. 3	NE	NE	4
Irving and Young [13] Case no. 1	8	NE	NE
Irving and Young [13] Case no. 2	10.5	NE	NE
Irving and Young [13] Case no. 3	12	NE	NE
0023904 [14-16]	11.5	26 days	16
0045156 [14-16]	9	4.5	17.7

As shown, the median time of diagnosis of ovarian metastases is absolutely unusual, which is 10.5 months after the detection of the primary tumor (-2 to 57 months) but the most important feature to highlight is the median OS which is 16 months (4-68 months). Except for this present case, other patients were treated with conventional chemotherapy regimens, which makes these data outstanding as the median survival in the extensive disease stage was 9-11 months [3]. We can hypothesize that the bloodstream is the preferred way of metastasization, in light of the absence of malignant cells in many peritoneal washing reported, and, therefore, the richly vascularized ovary of younger women (median age 43 years) is more prone to metastasis than in older patients. Sternberg also supposed that the ovary has a congenial environment for growth of metastasis and these favorable conditions may explain the development of single, large metastasis [17]. Ovarian metastases seem to mimic primary ovarian tumors with a big, mostly monolateral rather than small, bilateral diffusion and, despite the limited cases, sometimes expressing CA125, which could lead to a misdiagnosis. Although TTF-1 is not specific for SCLC as it is also expressed in adenocarcinomas of the lung and thyroid, it is a highly sensitive marker for SCLC of pulmonary origin as it is reported in the literature to have a very high frequency, hence it could be a keystone to distinguish SCLC of the lung from that of another origin [18]. SCLC is a subtype of lung cancer with a poor prognosis, and despite the innovative role of immunotherapy, the very modest PFS and OS benefits clearly emphasize the need for the identification of predictive biomarkers and more performing therapy. To date, there are no cases in the literature describing such an unusual clinicopathological presentation with an exceptional response to immunochemotherapy. A possible explanation for this great disease control could lie on the small tumor burden of the disease in this case. Given the only site of metastasization surgically removed and the integration of lung radiotherapy, we can speculate that this important result is secondary to a stage of disease attributable to the third (limited disease) compared to the fourth (metastatic disease). However, also comparing the expected outcomes of a third stage of SCLC, we have achieved a great result (median OS of 15-20 months with a two-year survival rate of 20-40%) [19]. Liu et al. [3] published updated OS data of Impower133 and they ran an exploratory analysis of efficacy based on PD-L1 expression levels. Despite the limited sample size, patients with PD-L1 ≥5%, as in our case, had a higher median OS (21.6 months) in the immunotherapy arm compared to control and other subgroups. Even if the role of PD-L1 is controversial and has to be further investigated, our assessment might help to validate in future a PD-L1 cutoff of response to immunotherapy. Furthermore, Gay et al. [20] have recently identified four subtypes distinguished by the differential expressions of transcription factors. The inflammatory subtype gained the greatest benefit from immunotherapy; although Impower133 was not statistically powered for subtype-specific subgroup analyses, they observed a median OS in the inflammatory type treated with chemo-immunotherapy of more than 18 months, compared to placebo and other subtypes. Interestingly, it seems to exhibit epithelial-mesenchymal transition and to be the most mesenchymal subtype which could explain the higher avidity for a stromal organ like an ovary. Taken together these considerations, it may not appear unfounded to hypothesize that the metastasization at the ovarian level may represent a positive predictor of the efficacy of the chemo-immune combination in the treatment of SCLC selecting a neoplasm that is particularly recognizable by the immune system. Finally, we resumed what is, to date, the state of the art about this very rare pathological condition trying to identify the common clinical characteristics and provide feasible explanations and a possible good prognostic role in this very aggressive tumor. To note, our report is the first in the literature to emphasize the role of ovarian metastasis as a predictive factor of response to immunotherapy.

## Conclusions

In this case report, we have detailed a unique instance of ovarian metastasis from SCLC demonstrating a remarkable and prolonged response to immunotherapy. By reviewing the literature from 1950 to the present, we have summarized other documented cases of ovarian metastases from SCLC, highlighting common clinical and pathological characteristics and differentiating these metastases from primary ovarian tumors. Additionally, we have explored the potential mechanisms underlying the durable response to immunotherapy observed in this case. As the literature on this tumor is growing together with the use of immunotherapy, we believe that our case, along with the review of all other published reports, may be relevant in order to provide some insight into the possible prognostic and predictive role of the spread in a tumor with a very poor prognosis.
